# The contribution of fire to the late Miocene spread of grasslands in eastern Eurasia (Black Sea region)

**DOI:** 10.1038/s41598-019-43094-w

**Published:** 2019-05-01

**Authors:** Angelica Feurdean, Iuliana Vasiliev

**Affiliations:** 1 Senckenberg Research Biodiversity and Climate Research Centre, Senckenberganlage 25, 60325 Frankfurt am Main, Germany; 2 Department of Geology, Babeş-Bolyai University, Kogalnicanu 1, 40008 Cluj-Napoca, Romania

**Keywords:** Fire ecology, Fire ecology, Grassland ecology, Grassland ecology, Palaeoecology

## Abstract

Grasslands are globally extensive, but the processes governing their ecology and evolution remain unclear. The role of fire for the expansion of ancestral C_3_ grasslands is particularly poorly understood. Here we present the first biomass combustion record based on late Miocene to Pleistocene (~10–1.9 Ma) charcoal morphologies (grass, herbs, wood) from the Black Sea, and test the extent of fire events and their role in the rise of open grassy habitats in eastern Eurasia. We show that a mixed regime of surface and crown fires under progressively colder and, at times, drier climates from the late Miocene to Pliocene (8.5–4.6 Ma) accelerated the forest to open woodland transition and sustained a more flammable ecosystem. A tipping point in the fire regime occurred at 4.3 Ma (mid-Pliocene), when increasingly cold and dry conditions led to the dominance of grasslands, and surface, litter fires of low intensity. We provide alternative mechanisms of C_3_ plant evolution by highlighting that fire has been a significant ecological agent for Eurasian grasslands. This study opens a new direction of research into grassland evolutionary histories that can be tested with fossil records of fire alongside climate and vegetation as well as with dynamic vegetation modells.

## Introduction

Grasslands have among the world’s highest species diversity, harbor endemic and threatened plant and animal species, and provide critical ecosystem services^1^. However, limited knowledge of the ecological processes governing grassland function and evolution has contributed to the loss of grasslands with cascading, negative consequences for biodiversity^1^. Research on the evolution and expansion of grasslands reveal that the rise of this ecosystem was a long process occurring asynchronously across continents^2–4^. For example, the ancestral C_3_ savanna mosaic spread during the mid to late Cenozoic (~55–33 Ma), whereas C_4_-dominated open habitats, better adapted to high temperatures, low water availability, and low CO_2_ concentration, expanded during the late Neogene (~10–8 Ma)^2,5^. In Europe, fossil plant records (pollen, macrofossils) show that open biome development started during the early to middle Miocene (~20 Ma), though full development occurred since the late Miocene (~10 Ma)^4^. In contrast, eastern Mediterranean phytolith remains (microscopic silica bodies abundantly produced in grass tissues and better preserved in drier environments) show that grass-dominated habitats were prevalent by the early Miocene (~20 Ma)^6^.

Several drivers have been put forward to explain forest loss and the expansion of an open grassy biome. Earlier hypotheses highlighted declining atmospheric CO_2_ concentrations and temperatures and increased aridity and seasonality of precipitation as the main drivers of grassland spread^5,7^. More recently, hypotheses have focused on the influence of local-scale factors such as fire and herbivores^4,8,9^. The critical role of fire in the rise of the savanna mosaic has been confirmed from fossil pollen, charcoal, and leaf wax *n*-alkanes records in C_4_-dominated grasslands in southern Africa^10^. However, whether variations in fire disturbances have contributed to the forest-grassland transition in Eurasian C_3_-dominated grasslands remains unclear. The involvement of fire is likely because the ancestral transition from forest to open grassy biome occurred in C_3_ species, but the rise of grasslands are explained based on processes that maintain tree-grass interactions in C_4_-dominated ecosystems^3^. Furthermore, recent studies from non-forested ecosystems (grasslands, shrublands, savannas) question the view of climate as the only driver of vegetation, and increasingly emphasises the role of disturbance by fire and herbivores^11,12^.

To test the hypothesis that fire contributed to the rise of C_3_-dominated open habitats in eastern Eurasia, we used the charcoal morphology record of biomass burning from the Deep Sea Drilling Program (DSDP) 42B 380 A core retrieved from the Black Sea. This 1075 m thick core represents the longest and most complete existing record of the late Miocene to Pleistocene (~10 to 1.9 Ma) Black Sea sedimentary and climatic history. Charcoal morphotypes (grass, forbs, wood) provide evidence of major changes in: i) the source of the fuel burnt (herbs vs. wood), ii) fire type (low intensity- surface vs. high-intensity crown fires), and iii) grassland community dynamics. Charcoal is one the most robust proxy of past fire frequency and fuel type, however, the amount of charcoal does not reflect the fire history per se, but how charcoal resulting from fires is produced, transported, and incorporated into sediments^13^. Our study is the first approach to quantify the long-term extent of fires in temperate open habitats of Eurasia, providing a new view on the ecology and evolution of C_3_ grasslands independent of the C_4_ pathway.

## Results and Discussion

### Evidence for changes in sediment provenance and charcoal

**Figure 1:**
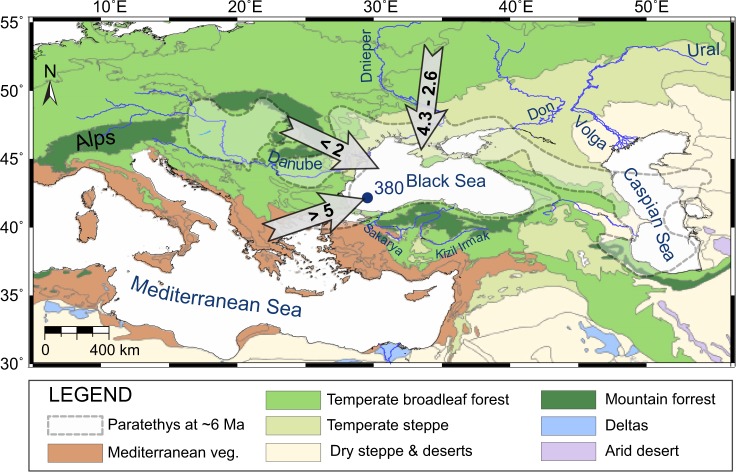
Sediment transport into the Black Sea. Major rivers draining into the Paratethys highlight major sediment transport routes^26^. Arrows show the dominant direction of sediment transport into the Black Sea during the assigned ages^16^ whereas the bold numbers denote Ma. The location of DSDP Site 380/380 A in the Black Sea is indicated by the blue dot labelled ‘380’, along with modern vegetation distribution in the entire region.

**Figure 2:**
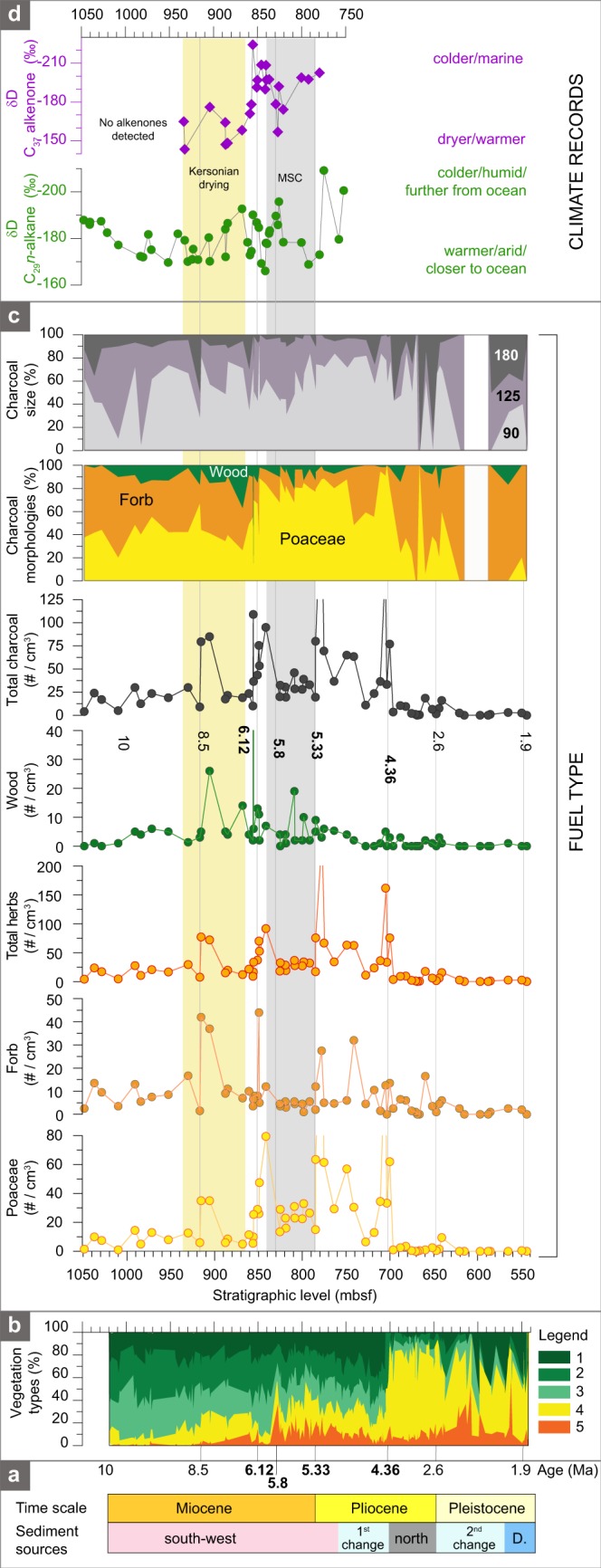
Biomass burning, fuel type, vegetation, and climatic conditions during the Miocene to early Pleistocene (~10–2 Ma). (**a**) Changes in sediment provenance;^16^ ‘D’ is Danube) are shown within the chronological framework of this study. (**b**) The relative abundance of five ecological groups based on the pollen record:^20^ 1 = subtropical forest, 2 = warm temperate forest, 3 = coniferous forest, 4 = herbs, and 5 = steppe elements. (**c**) Biomass burning reconstructed from total charcoal concentrations and percentages and fuel type and fire intensity based on charcoal morphologies (Poaceae, forbs, wood). Total herbs sums Poaceae and forb morphotypes. Poaceae and forb morphologies originate from burning by low-intensity surface fires, whereas wood morphology primarly originates from severe burning of the tree crown. Charcoal class size (90, 125 and 180 μm) is given in percentages. (**d**) Climatic conditions derived from the hydrogen isotopic compositions (δD) of C_29_ long-chain *n*-alkanes and C_37_ alkenones (an evaporation proxy) from the same DSDP core^24^. ‘MSC’ denotes the Messinian Salinity Crisis.

Charcoal arriving in marine environments follows two main routes: atmospheric deposition in the case of smaller particles and riverine discharge for small and large particles alike^14,15^. Today, the Danube provides 53% of the fresh water and sediment discharge into the Black Sea, the Dnepr and Don provide about 30%, and rivers flowing from the south and southeast ~4% (Fig. 1). However, source-to-sink analysis shows that the provenance of Black Sea sediments 1050–755 m below sea floor (mbsf) (~10–5 Ma) was predominantly from southerly to southwesterly sources such as the Balkan and Anatolian rivers (Fig. 2a)^16^. A transition to a northerly Russian Platform source with episodic delivery of sediments from the Danube occurred between ~5 and 4.3 Ma (755–708 mbsf), and sediments were sourced almost solely from the Russian Platform between ~4.3 and 2.6 Ma (708–651 mbsf). The influx of Danube-supplied sediment to the southwestern Black Sea began after ~4.3 Ma^16^, however, the final arrival of Danube sediments into the Black Sea took place during the Pleistocene i.e., 571 mbsf (~2 Ma)^16–18^.

### Evidence for changes in fire regimes and vegetation communities

The charcoal record of DSDP 42B 380 A shows an increase in total charcoal concentration from ~0–25 pieces/cm^3^ at the base of the core (1050 mbsf, ~10 Ma) to maximum values in the profile (~50–250 pieces/cm^3^) between 920 and 700 mbsf (~8.5–4.3 Ma), and a subsequent decline of charcoal abundance to minimum values in the record (~0–5 pieces/cm^3^) between 700 and 540 mbsf (~4.3–1.9 Ma; Figs 2c and S2). Poaceae and forb (herbs) charcoal morphotypes are co-dominant between 1050 and 920 mbsf (~10–8.5 Ma), herbaceous (70–90%) and woody charcoal (10–30%) are mixed between 920 and 750 mbsf (~8.5–5 Ma), and charcoal became almost exclusively of herbaceous type between 700 and 540 mbsf (~4.3–1.9 Ma; Figs 2c and S2). Charcoal become smaller in size from the base (~10 Ma) to ~4.3 Ma, and become larger again between ~4.3 and 1.9 Ma (Fig. 2). Peak charcoal concentrations of notably high magnitude and large particle size are at 850, 785, and 701 mbsf (~6.1, ~5.33, and ~4.3 Ma, respectively; Fig. 2c). The peak at ~6.1 Ma corresponds to Pontian flooding in the Paratethys, a time of marine ingress from the Mediterranean Sea^19^. The peak at ~5.33 Ma closely coincides with Pliocene flooding, when Atlantic waters invaded the Mediterranean Sea and briefly reached the Black Sea^19^. Only the peak at 701 mbsf corresponds to a change in sediment provenance (Fig. 2a,c).

The pollen-based vegetation reconstruction^20^ indicates stepwise changes in main ecological groups (Fig. 2b). The percentages of herbs (Poaceae, Asteraceae, Brasicaceae, etc.) and steppe elements (*Artemisia* and *Ephedra*) were low (~10%) until 940 mbsf (~8.5 Ma), whereas those of subtropical, warm temperate and coniferous forests show an almost equal representation. The percentages of herbs (grasses and forbs) and steppic plants rose to ~20–30% between 940 and 700 mbsf (~8.5–4.3 Ma), coincidently to the decline in all forest types (Fig. 2b). Herbaceous pollen percentages increased abruptly to ~80% at 700 mbsf (~4.3 Ma) and remained dominant until 540 mbsf (~1.9 Ma),

Two main patterns in the dynamics of the dominant fuel type burnt and the development of grass and forbs habitats are apparent in the charcoal morphotype record from the Black Sea core (Fig. 2c). First, we recorded the greatest biomass burning and fire of high- intensity when mixed herbaceous and woody fuels were prevalent during the late Miocene (~8.5 Ma) to the early and middle Pliocene (~5.33–4.3 Ma). Biomass burning declined markedly and fire were of low- intensity from the late Pliocene to the early Pleistocene (~4.3–1.9 Ma) when fuel originated predominantly from herbaceous plants (Fig. 2b,c). Second, there is a strong association between increased biomass burning, particularly of grass (Poaceae) fuel, at ~8.5 Ma and the rise in dominance of grass and forb habitats as inferred from pollen records (Figs 2b,c and S2). The charcoal and pollen records thus provide strong evidence for a shift from mixed surface and crown fires during the transition from closed forests to open woodlands during the late Miocene and early Pliocene (~8.5–4.3 Ma) towards surface fires when open grassy biomes dominated during the Pliocene to early Pleistocene (~4.3–1.9 Ma).

### The link between fire and vegetation turnover: implication for grassland expansion

Although grasslands are poorly competitive against trees, they tolerate soils with permanent or seasonal moisture deficits, temperature extremes, and disturbances by fires and herbivores, which otherwise limit the establishment and growth of woody plants^21^. It has been shown that the global tendency towards cool and arid conditions that characterised the late Miocene gave rise to an open biome^22,23^, although the extension of grass-dominated habitats in the Mediterranean region has been demonstrated to occur as soon as the early Miocene^4^. The onset of the expansion of open habitat vegetation from ~8.5 to 5.97 Ma in the Black Sea region was concurrent with slightly enhanced biomass burning (Fig. 2b–d) and largely increasing evaporative conditions in the Black Sea basin, as indicated by the alkenone-based hydrogen isotopic (δD) record (an evaporation proxy) measured from the same core^24^. The presence of carbonate nodules requiring strong climate seasonality show that evaporative conditions affected the entire circum-Black Sea region around 8.5 Ma^25–28^. This part of the sedimentary sequence is primarily sourced from the Balkans and Anatolia, although charcoal fragments transported by water and air could have originated from the entire Black Sea region (Fig. 1). A pytholith-based vegetation reconstruction from Turkey indicates a C_3_-grass-dominated savanna mosaic at that time^6^. Similarly, stable carbon isotopic analyses (δ^13^C) of palaeosols from the Greco-Iranian region and pollen records from Bulgaria indicate the development of open vegetation in the late Miocene^6,29^. Further support for the progressive drying of the late Miocene environment (~8–5.3 Ma) around the Black Sea and Eastern Mediterranean is found in the rise of large mammals adapted to open habitats^30,31^. It is therefore probable that the warm and especially dry climatic conditions that prevailed during large intervals between ~8.5 and 5.97 Ma around the Black Sea may have led to the drying of fuels in the subtropical-warm temperate deciduous-coniferous forests and promoted mixed surface and crown fires. An increasing proportion of herbaceous plants in formerly closed forests may have provided a favorable fuel mix, i.e., fine herbs and coarse wood, therefore escalating ignition potential, flammability, and fire spread^32^.

Climatic conditions became increasingly dry and temperatures declined during the late Miocene to middle Pliocene (~5.97–4.3 Ma), especially in the northern Black Sea^24,26,27,29^. Though fluctuating, biomass burning peaked and the more frequent fires likely promoted grassland expansion, accelerated the forest to open woodland transition and sustained a more flammable ecosystem (Figs 2b,c and S2). Studies of grass evolution in tropical savannas (C_4_-type) indicate that their fine fuels with rapid curing, low bulk densities, and fast regrowth rates led to frequent surface fires, and that woody species, adapted to infrequent fires, were unable to complete their life cycle^5,9,33,34^. Sediment provenance analysis suggests continued sediment transport from the Balkan and Anatolian rivers (Fig. 1), thus areas increasingly covered by open vegetation, although this source becomes uncertain towards the later part of this interval, i.e., ~5.33–4.3 Ma (Fig. 2a). Pollen records from northern regions (i.e., the Ukrainian Plain) corroborate an increasingly cold and dry climate along a west-to-east gradient during this time, and vegetation dominated by forest-steppe and open xerophytic herbs^29^. It is noteworthy that the sustained decline in biomass burning from ~5.8 to 5.33 Ma was coeval with the Messinian Salinity Crisis (Fig. 2d), a period characterised by a large disruption in the regional hydrological cycle, a major water level drop in the Mediterranean Sea^35^, and enhanced northern hemisphere glaciation that culminated during the TG 20 and TG 22 glacial peaks^36^.

A tipping point in the fire regime occurred at ~4.3 Ma and the new established fire regime lasted at least until the end of our record (~1.9 Ma, early Pleistocene). It is evident as a decrease in burning activity, the predominance of herbaceous charcoal fuel and the maximum extent of grassy vegetation (Figs 2b,c and S2). A regional differentiation in climate, vegetation, and fauna assemblages was apparent between ~4.2 and 2 Ma, when Central and Eastern Europe (the major source feeding sediments into the Black Sea at that time) remained arid, while the Mediterranean region became more humid^23,30,37^. We propose that low vegetation productivity and thus fuel availability of grassy vegetation limited the biomass burned yet, surface fires were perhaps frequent. Low amounts of burned biomass and/or frequent fires have been documented in modern low productivity grassy environments and during past dry and cold climatic periods of slow vegetation growth and fuel accumulation^15,38–41^. Herbivores are also known to remove vegetation load, leading to reduced fuel availability and therefore biomass burned^42^. By ~15 Ma, ca. 25% of the world’s mammals were typically hypsodont grazers and 35% of mixed diet^43^, whereas hypsodont grazers rose to dominance in Eurasia from ~8 to 4.5 Ma^23,37^. It is therefore probable that the observed decline in biomass burning may have also been a response to increased grazing pressure and reduced fuel availability.

We further tested whether periods of high fire occurrence coincided with increased representation of C_4_ plants in the surrounding vegetation, as this vegetation type has a strong competitive advantage under frequent fires^5,44^. In eastern Eurasia, C_3_ plants dominated throughout the Neogene, whereas C_4_ grasses were of little ecological importance^6,7^. The δ^13^C values of C_29_ long-chain *n*-alkanes from the same Black Sea record covering the period ~10–5 Ma are in the range of C_3_ plant communities (−27 to −30‰; Fig. S2). This indicates that the enhanced late Miocene fire activity was associated with the dominance of C_3_ grasses and further highlights that fire has been a significant ecological agent for C_3_ plants in the Black Sea region. Simulated late Miocene vegetation scenarios for Africa, show that fire allowed both C_3_ and C_4_ grasses to expand into forests^9^.

## Conclusion

We provide a unique charcoal morphotype record of the role of fire disturbances in the spread and evolution of temperate grassy ecosystems in Eurasia and identify mechanisms that controlled biomass burning in the Black Sea region over six million years. Results show that progressively colder and episodic dry climatic conditions from the late Miocene to middle Pliocene (~8.5–4.3 Ma) promoted fire activity, accentuated the forest-grassland transition, and sustained a more flammable ecosystem. A tipping point in the fire regime occurred at ~4.6 Ma, when the dominance of open grassy vegetation limited biomass productivity and fuel availability and connectivity, leading to surface, low intensity, but likely frequent fires. As the transition to this new vegetation state (grasslands) was associated to an abrupt shift in fire regime, it suggests thresholds in tree-grass cover which, when crossed, resulted in shifts in fire regime. Our results provide an alternative view on grassland ecology and evolution by showing that fire has been a significant ecological and evolutionary agent in temperate C_3_-plant-dominated open habitats in Eurasia. Critically, we provide a new framework for better understanding the evolution of temperate grasslands and testing new predictions using fossil charcoal records alongside those for climate and vegetation, as well as dynamic vegetation modells.

## Methods

The DSDP 42B 380 A core (42° 05.94′N, 29° 36.82′E) represents a 1075-m-thick late Miocene to Quaternary (~10 to 0.5 Ma) sedimentary record from the Black Sea^45^. The construction of geochronological framework was performed through combined magnetostratigraphy and ^40^Ar/^39^Ar dating^46,47^ as well as novel biomarker geochemistry and pollen records^24,48,49^. [See Fig. S1].

### Charcoal-based reconstruction of biomass burning and grassland dynamics

We estimated changes in regional biomass burning in the Black Sea region by analysing sedimentary charcoal in 65 levels of approximately 2 cm^3^ each from the 1075–550 mbsf section, representing the late Miocene to the Pliocene (~10 to 1.9 Ma). Each sample was bleached and wet sieved to 90, 125, and 180 μm respectively, resulting in a total of 195 samples. Sedimentary charcoal pieces were categorised into three main morphotypes (grasses, forbs and wood). The size class and morphotype separation allows us to: a) better constrain the charcoal source area^50,51^, where the finer particles (90 and 125 μm) are sourced farther away than the larger particles (180 μm), and b) provide additional information on the fuel source and fire severity, where grass and herb morphotypes primarily originate from low-intensity surface fires and wood from high-intensity crown fires^51–56^. Crown fires also supply larger charcoal particles and in greater amounts than surface herbaceous fires^51,57^. The remaining material was transferred to a Petri dish, counted, and examined under a stereoscope at 30–60X magnification. Opaque, rectangular particles were classified as charcoal, and some had preserved anatomical structures. The source of the main morphotype categories are: 1) grasses (Poaceae), 2) forbs resulting from the burning of herbaceous plants other than grasses, and 3) wood (ligneous material) following previously described methodologies^51–56^. We note the rare occurrence of other morphologies such as roots (charcoal likely derived from roots, stolons, rootlets) and deciduous leaves. The resulting macroscopic charcoal counts are expressed as the concentration of total particles (particles/cm^3^) and the concentration of each charcoal morphotype by dividing the counts by the sediment volume (Fig. 2c). We calculated percentages of each charcoal morphotype and size using the sum of charcoal counts (Figs 2 and S2).

Theoretical functions and calibrations for the relationship between fire and charcoal production and deposition in sedimentary basins suggest that increased charcoal levels may correlate with fire severity (fuel consumption per fire episode)^58,59^, and the size of the area burnt at the ecosystem scale^13,60^. Distance from the fire source, charcoal transport and deposition can also affect charcoal accumulation 13,60]. Therefore our reconstructed rise in biomass burning integrates information about the increase in area burnt, severity of fire events but also the proximity of the fire source and transportation.

### Pollen-based reconstruction of vegetation

To determine past vegetation dynamics we used a published pollen-based vegetation record from the same core^20^. We extracted five main ecological groups following the classification of^20^. These are: 1 = subtropical forests (*Buxus colporate Canthium*, Acanthaceae, Sapindaceae, Sapotaceae, *Bombax*, Taxodiaceae, Arecaceae, *Engelhardia*, *Platycarya*, *Distylium*, *Hamamelis*, *etc*); 2 = warm-temperate forests (*Quercus*, *Carya*, *Pterocarya*, *Carpinus*, *Ulmus*, *Tilia*, *Alnus*, *Betula*, *etc*); 3 = coniferous forests (*Pinus*, *Picea*, *Abies*, *Tsuga*, *Cedrus*); 4 = herbs (Poaceae, Asteraceae, Brasicaceae, Lamiaceae, Chenopodiaceae, etc); and 5 = steppe elements (*Artemisia* and *Ephedra*).

## Supplementary information

SI 1

## Data Availability

The charcoal datasets will be made available online and deposited into PANGEA.
