# Supplementary material for: The contribution of fire to the late Miocene spread of grasslands in eastern Eurasia (Black Sea region)
Source: Sci Rep. 2019 May 1;9:6750. doi: 10.1038/s41598-019-43094-w (PMC6494819; doi:10.1038/s41598-019-43094-w)
Supplement: Supplementary file 1 — SI 1 [file 41598_2019_43094_MOESM1_ESM.pdf]

Supplementary Material: The contribution of fire to the late Miocene spread of grasslands in eastern Eurasia (Black Sea region) by Angelica Feurdean and Iuliana Vasiliev

Chronostratigraphy

DSDP core 42B 380A was drilled in 1975 and encompasses approximately the last ten million years, representing the longest known record of past environmental change in the Black Sea region. The construction of a reliable age model at the time of drilling was hampered by the absence of foraminifera and nanofossil groups commonly used for stratigraphic correlation. Recently, this record was integrated into a solid geochronological framework through combined magnetostratigraphy and $^{40}Ar/^{39}Ar$ dating [1,2] as well as novel biomarker geochemistry and pollen records [3,4,5]. The DSDP 42B 380A geological record is constrained by the Pontian flooding event, marking the end of the Maeotian (regional Paratethys stages), dated at ~6.12 Ma (~855 mbsf) and by an ash layer dated at 4.3±0.19 Ma (706 mbsf; Fig. S1). This new geochronology pins the Miocene-Pliocene boundary at ~5.33 Ma (~785 mbsf; [1]. The exact chronology of the interval of the core older than 6.12 Ma (~855 mbsf) remains uncertain. However, according to [6,7], that interval covers the Upper Miocene, and most likely the Bessarabian and Khersonian Paratethys regional sub-stages; the pebbly mudstone unit may indicate deep desiccation of the Black Sea during late Khersonian regression (~8–9 Ma). Bearing in mind that this interval is marked by drying in the $\delta D_{alkenone}$ record [5,8], we consider that all observations converge towards an age of ~8.5 Ma for the level at 930 mbsf (Fig. S1). The chronology of the upper part of the core, younger than 4.3 Ma (<706 mbsf) is quasi-constrained (Fig. S1). From the three options presented by [2] we favour their option B, in which the magnetic reversal at 640 mbsf correlates to the top of the Gauss chron (2.581 Ma) and allows correlating the overlying reverse zone to chrons C1r and C2r. In this case, the interval between 640 and 700 mbsf should span the time interval 2.58–4.35 Ma. The first option is to use a lower sedimentation rate and correlate zone N5 to chron C2An (Gauss) and N6 to C2Ar (Option B1). Alternatively, Option B2 calls for a hiatus at 700 mbsf and correlates N5 to chron C2An.1n (top of the Gauss). For the interval above the hiatus, this would imply a relatively constant sedimentation rate of ~11 cm/kyr, similar to that below 700 mbsf. The total duration of the hiatus at 700 mbsf would be ~1.3 Myr. More arguments for assigning an age of ~2.6 Ma to the 640 mbsf level are provided by the coupled provenance analyses of [9,10,11]. Source-sink analysis [9] suggests that Danube sediment arrived in the Black Sea during the Pleistocene. This is in good agreement with the geology of the eastern Dacian Basin, which became a predominantly fluvial environment during the regional Romanian

stage (4.15–1.8 Ma; [1]). These observations are in line with the finding that the Pannonian Basin trapped all Danube sediments until at least 4 Ma ago [12].

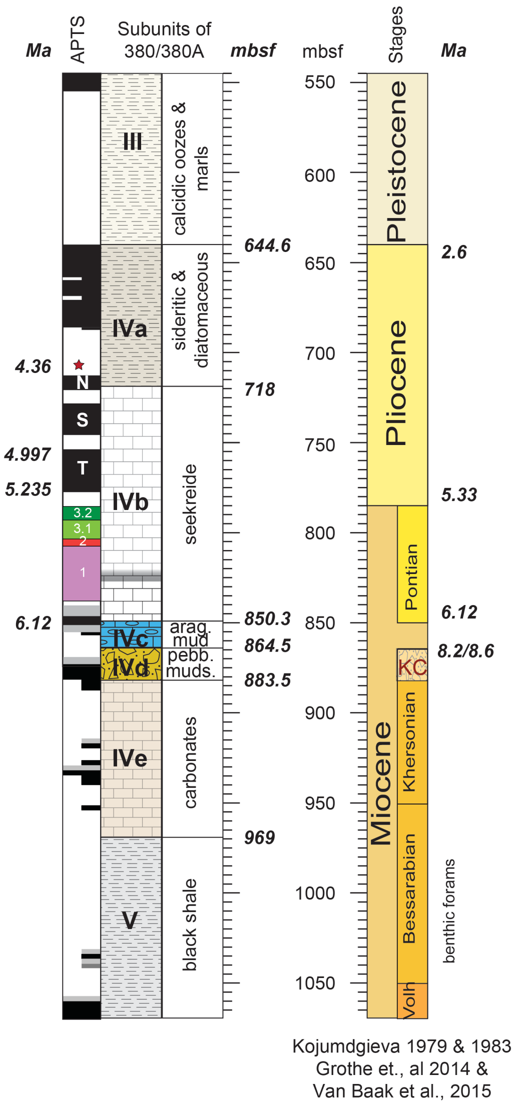

Figure S1. Chronostratigraphy of the Miocene to lower Pliocene succession in DSDP core 42B 380A. APTS represents Astrronomically Tuned Polarity Time Scale [13]. The red star denotes an ash layer dated to 4.3±0.19 Ma. The paleomagnetic polarity column (left) was correlated to the Astronomically Tuned Polarity Time Scale (APTS) [13]. T indicates Thvera normal polarity chron with written lower and upper ages next to the magnetostratigraphy. 1, 2, 3.1 and 3.2 are indicatives for different stages of the MSC: Stage 1 (5.97–5.6 Ma), Stage 2 (5.6–5.55 Ma), Stage 3.1 (5.55–5.42 Ma) and Stage 3.2 (5.42–5.33 Ma) [14]. The sampled interval covers 7 lithological (sub)units [15]: unit V consisting of black shale (1072.5 – 969.0 mbsf), unit IVe of 'carbonate' (969.0 – 883.5 mbsf), unit IVd of pebbly mudstone (883.5 – 864.5 mbsf), unit IVc of aragonitic mud (864.5 – 850.3 mbsf); unit IVb, the so-called 'Seekreide', (850.3 – 718.0 mbsf, unit IVa of sideridic and diatomaceous marls; unit III 850.3-550 mbsf of calcitic oozes and marls). At 6.12 Ma the Pontian flooding event occurred. The red star denotes an ash layer dated to 4.36±0.19 Ma [1]. KC represents the interval of the Khersonian Crisis, a drying event affecting circum-Black Sea area. In the time scale (right) Volhinian (Volh), Bessarabian, Khersonian and Pontian are regional Paratethys substages, all part of the upper Miocene.

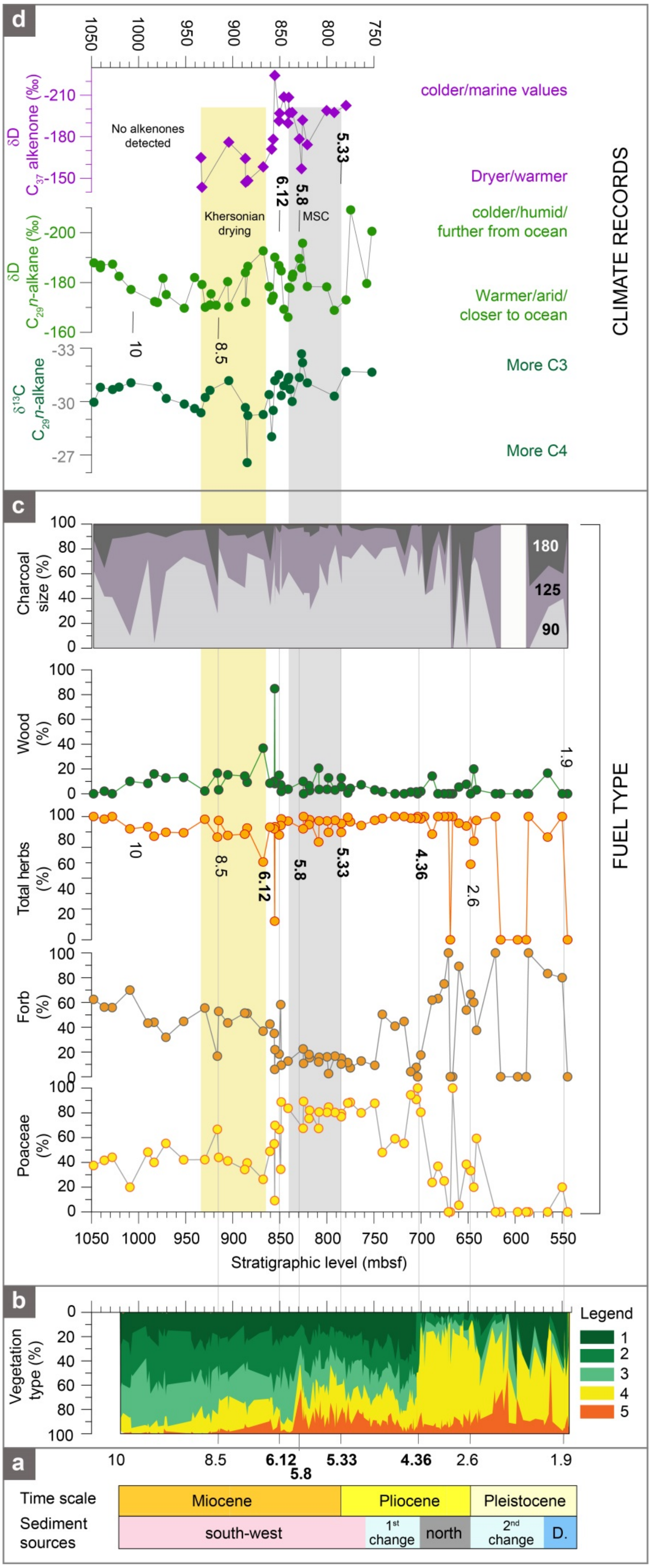

Figure S2. Biomass burning, fuel type, vegetation, and climatic conditions during the Miocene to early Pleistocene (~10–2 Ma). a) Changes in sediment provenance [11] ('D' is Danube) are shown within the chronological framework of this study. b) Vegetation is separated into five main ecological groups: 1 = subtropical forests; 2 = warm-temperate forests; 3 = coniferous forests; 4 = herbs; and 5 = steppe elements following the classification of [16]. Since the data sets are not available as raw counts, we could not enumerate the pollen percentages of Poaceae separately from other herbs. c) Selected charcoal morphologies and class sizes presented as percentages. d) Climatic conditions are derived from the carbon and hydrogen isotopic compositions of C_{29} long-chain *n*-alkanes and the hydrogen isotopic compositions of C_{37} alkenones [5]. The $\delta^{13}C_{n\text{-alkane}}$ values covering the period ~10–5 Ma show the prevalence of C_3 plant communities in the range from –27 to –30‰.

References

1. van Baak, G.C. *et al.* Messinian events in the Black Sea. *Terra Nova* **27,** 433–441 (2015).

2. van Baak, G.C *et al.* Objective utilization of data from DSDP Site 380 (Black Sea).*Terra Nova* **28**, 230-231 (2016).

3. Popescu, S.M. Late Miocene and early Pliocene environments in the southwestern Black Sea region from high-resolution palynology of DSDP Site 380A (Leg 42B). *Palaeogeography, Palaeoclimatology, Palaeoecology*, **238**, 64–77 (2006).

4. Grothe, A. *et al.* Black Sea desiccation during the Messinian Salinity Crisis: Fact or fiction? *Geology* **42**, 563–566 (2014).

5. Vasiliev, I. *et al.* Recurrent phases of drought in the upper Miocene of the Black Sea region. *Palaeogeography Palaeoclimatology Palaeoecology* **423**, 18–31 (2015).

6. Kojumdgieva, E. 1979. Critical notes on the stratigraphy of Black Sea boreholes (Deep Sea Drilling Project, Leg 42B): *Geologica Balcanica* **9**, 107–110.

7. Kojumdgieva, E. 1983. Palaeogeographic environment during the desiccation of the Black Sea. *Palaeogeography, Palaeoclimatology, Palaeoecology* **43,** 195–204.

8. Vasiliev, I., Reichart, G.J. & Krijgsman, W. Impact of the Messinian Salinity Crisis on Black Sea hydrology — insights from hydrogen isotopes on molecular biomarkers. *Earth and Planetary Science Letters* **362**, 272–282 (2013).

9. Maynard, J.R., Ardic, C. & McAllister, N. *Source to sink assessment of Oligocene to Pleistocene sediment supply in the Black Sea*. In N. C. Rosen, et al. (Eds.), 32nd Annual GCSSEPM Foundation Bob F. Perkins Research Conference. New understandings of the petroleum systems of continental margins of the worldHouston, Texas, 27. (2012).

10. Olariu, C, Krezsek, Cs. & Jipa. DC. The Danube River inception: Evidence for a 4 Ma continentalscale river born from segmented ParaTethys basins. *Terra Nova* **9**, 1–9 (2017).

11. de Leeuw, A, Morton, A., van Baak, C.G.C. & Vincent, S.J. Timing of arrival of the Danube to the Black Sea: Provenance of sediments from DSDP site 380/380A. *Terra Nova* **30**,114–124 (2018).

12. Magyar, I. *et al.* Progradation of the paleo-Danube shelf margin acrossthe Pannonian Basin during the Late Miocene and Early Pliocene. *Global and Planetary Change*, **103**, 168–173 (2013).

13. Lourens, L.J., Hilgen, F.J., Laskar, J., Shackleton, N.J. & Wilson, D.S. The Neogene period. In: Gradstein, F., Ogg, J., Smith, A. (Eds.), A Geological Timescale. Cambridge University Press. 409–440 (2004).

14. Roveri, M. *et al*. The Messinian salinity crisis: past and future of a great challenge for marine sciences. *Marine Geology* **352**, 25–58 (2014).

15. Ross, D.A. & Neprochnov, Y.P. Initial reports of the Deep Sea Drilling Project. Vol. 42, Part 2 *U.S. Government Printing Office*, Washington (1978).
16. Popescu, S.M. *et al*. Pliocene and lower Pleistocene vegetation and climate change at the European scale: long pollen records and climatostratigraphy. *Quaternary International* **219**, 152–167 (2010).
